# P120-Catenin Regulates Early Trafficking Stages of the N-Cadherin Precursor Complex

**DOI:** 10.1371/journal.pone.0156758

**Published:** 2016-06-02

**Authors:** Diana P. Wehrendt, Fernando Carmona, Ana E. González Wusener, Ángela González, Juan M. Lázaro Martínez, Carlos O. Arregui

**Affiliations:** 1 Instituto de Investigaciones Biotecnológicas, (IIB-INTECH), Universidad de San Martín, San Martín, Argentina; 2 Departamento de Química Orgánica, Facultad de Farmacia y Bioquímica, Universidad de Buenos Aires, CABA, Argentina; University of Illinois at Chicago, UNITED STATES

## Abstract

It is well established that binding of p120 catenin to the cytoplasmic domain of surface cadherin prevents cadherin endocytosis and degradation, contributing to cell-cell adhesion. In the present work we show that p120 catenin bound to the N-cadherin precursor, contributes to its anterograde movement from the endoplasmic reticulum (ER) to the Golgi complex. In HeLa cells, depletion of p120 expression, or blocking its binding to N-cadherin, increased the accumulation of the precursor in the ER, while it decreased the localization of mature N-cadherin at intercellular junctions. Reconstitution experiments in p120-deficient SW48 cells with all three major isoforms of p120 (1, 3 and 4) had similar capacity to promote the processing of the N-cadherin precursor to the mature form, and its localization at cell-cell junctions. P120 catenin and protein tyrosine phosphatase PTP1B facilitated the recruitment of the N-ethylmaleimide sensitive factor (NSF), an ATPase involved in vesicular trafficking, to the N-cadherin precursor complex. Dominant negative NSF E329Q impaired N-cadherin trafficking, maturation and localization at cell-cell junctions. Our results uncover a new role for p120 catenin bound to the N-cadherin precursor ensuring its trafficking through the biosynthetic pathway towards the cell surface.

## Introduction

Cadherins belong to a superfamily of transmembrane cell–cell adhesion molecules which play important roles in development, morphogenesis, and cancer [1, 2]. The function of cadherins is exerted at the cell surface, where extracellular domains of identical cadherins interact in a homophilic, Ca^+2^-dependent manner to form adherens junctions between adjacent cells. The intracellular domains interact with several cytoplasmic proteins, the most prominent of which are the catenins [3]. Proximal and distal regions of cadherin cytosolic domains interact directly with p120 catenin and β-catenin (or its close relative plakoglobin), respectively. Catenins bound to surface cadherins modulate cell-cell adhesion through different mechanisms involving cadherin recycling, stability, and coupling to the actin cytoskeleton. P120 binds to a ~40 amino acids region at the juxtamembrane domain of cadherins, masking clathrin-dependent endocytic motifs [4–7]. Therefore, p120 plays a key role as an inhibitor of cadherin turnover and as a "set point" for cadherin expression levels [8, 9]. Most cells express multiple p120 isoforms, and N-terminal splicing events lead to the use of four alternative start codons [10]. All isoforms contain a conserved and central Armadillo repeat domain which mediates equivalent binding to cadherin [11]. However, the efficiency in stabilizing cadherin at the plasma membrane differs among isoforms containing (isoform 3) or lacking (isoform 4) the N-terminal regulatory domain [12]. Thus, different p120 isoforms may affect cadherin function by recruiting distinct binding partners to the cadherin complex.

Cadherins biosynthesis occurs at ER-bound ribosomes as precursors containing a pro-domain at the N-terminus that inhibits cadherin dimerization and adhesion [13–15]. In a late Golgi compartment, the pro-domain is cleaved by pro-protein convertases of the furin family [15–17]. Beta catenin and p120 bind to the cytoplasmic domain of cadherin precursors, trafficking as a complex towards the cell surface [17–20]. The functional significance of catenin binding at this early stage of cadherin synthesis is unclear. P120 has been implicated in post-Golgi trafficking of cadherins to the cell surface via association and recruitment of the microtubule-associated motor kinesin [21]. Kinesin binds to the p120 N-terminal regulatory domain. Whether p120 bound to the N-cadherin precursor plays a role at earlier stages of anterograde trafficking has not been addressed. Cells expressing N-cadherin with the p120 binding site mutated, displayed accumulation of the precursor suggesting this possibility [22]. However additional cadherin partners, such as presenilin-1, ankyrin-G and the glutamate receptor interacting protein (GRIP), whose binding sites overlap with that of p120, could also be implicated [23–25]. In the present paper we specifically inhibited p120 expression by shRNAi and confirmed the role of p120 as a positive regulator of the anterograde traffic and processing of the N-cadherin precursor. We also show that p120 and protein tyrosine phosphatase PTP1B are required for recruiting N-ethylmaleimide sensitive factor (NSF) to the cadherin precursor complex. NSF is an essential ATPase of the vesicular trafficking machinery. It disassembles cis v/t-SNARE complexes maintaining free v- and t-SNARE pools necessary for membrane fusion events [26]. Our results suggest a novel mechanism by which p120 and PTP1B ensures the trafficking of nascent N-cadherin precursor through early stages of the biosynthetic pathway, likely implicating the NSF machinery.

## Materials and Methods

### Antibodies and reagents

Monoclonal antibody against N-cadherin was from BD Transduction laboratories (final dilution 1/4000). Monoclonal anti-p120 antibodies (clones 6H11 and 15D2; final dilution 1/1000) were kindly provided by A. B. Reynolds (Vanderbilt University, Nashville, U. S.). Monoclonal antibodies against HA (clone HA-7; final dilution 1/1000) and anti-α-tubulin (clone DM1A, final dilution 1/20000), and the polyclonal antibody against calnexin (final dilution 1/1000), were from Sigma-Aldrich. Monoclonal anti-NSF was kindly provided by T. Mustelin (Burnham Institute, La Jolla, U. S.) or obtained from Enzo Life Sciences (clone 9G7-3) or Abcam (clone NSF-1); all used at a final dilution 1/1000. Monoclonal anti-phosphotyrosine (clone 4G10, final dilution 1/750) was from Millipore. Alexa-488, Alexa-568 and Alexa-647 conjugated fluorescent secondary antibodies were from Molecular Probes (final dilution 1/1000). HRP-conjugated antibodies (final dilution 1/20000) were from Jackson Immunoresearch. Fluorescent secondary antibodies IR680 anti mouse and anti rabbit for infrared imaging were from LI-COR (final dilution 1/20000). Protein-G conjugated to Sepharose was from GE Healthcare and protein A/G conjugated to magnetic beads was from Thermo Scientific (final dilution 1/20000) Coverslips were from Marienfeld GmbH & Co and fibronectin was from Sigma-Aldrich.

### DNA constructs

N-cadherin with EGFP added at the C-terminal and the HA-tag inserted in the propeptide (pro-WT) and N-cadherin-3A mutant with YFP at the C-terminal and the HA-tag inserted in the propeptide (pro-3A), were previously described (HA-N-cadherin-EGFP and HA-N-cadherin-3A-YFP respectively, [22]). For colocalization studies we prepared pro-WT2 (pro-WT without EGFP) using pro-WT as PCR template (Forward primer: 5’ GGGAT ATCTGCCACCATGTGCCGGATAG 3’; Reverse primer: 5 ‘GCTCTAGATCAGTCAT ACCTCCACCGTACATGTC 3’). The PCR product was inserted into EcoRV/XbaI sites of the pcDNA3.1 plasmid. The cDNA of p120 isoforms 1A and 3A in pms vector were kindly provided by A. B. Reynolds (Vanderbilt University, Nashville, USA). For expression in SW48 cells, the isoforms were cloned into the EcoRI/EcoRV sites of pcDNA3.1. The isoform p120-4A was obtained by PCR, using p120-1A-pms as template, and cloned into the EcoRI/EcoRV sites of pcDNA3.1 (Forward primer 5´ CGGAATTCGTATGATTGG TGAAGAGGTCC 3´; Reverse primer: 5´ CCGATATCGGTACCAGGTCTAG 3´). All constructs were verified by DNA sequencing. The cDNAs of NSF-WT and NSF-E329Q in pcDNA3.1 were kindly provided by M. G. Coppolino (University of Toronto, Toronto, Canada). GalNacT-DsRed was kindly provided by J. L. Daniotti (Universidad Nacional de Córdoba, Córdoba, Argentina). EGFP-ERGIC-53 was a gift from H. P. Hauri (Biozentrum, University of Basel, Switzerland).

### RNA interference

For human p120 silencing by short hairpin interference RNA, an oligoduplex was obtained by hybridization of single strand oligonucleotides consisting of the sense and antisense target sequence of human p120 (underlined) separated by a 9 nucleotide loop: Sense, 5´ GATCCCCGCCAGAGGTGGTTCGGATATTCAAGAGATATCCGAACCACCTCTGGCTTTTT 3´; antisense, 3´ GGGCGGTCTCCACCAAGCCTATAAGTTCTCTATAGGC TTGGTGGAGACCGAAAAATCGA 5´. The oligoduplex was inserted into BglII/HindIII sites of pG-Shin2 vector (kindly provided by S.I. Kojima, Gakushuin University, Japan; [27]). A control plasmid was made inserting a target sequence of mouse p120 (5´ GCCAGAGTGGTGCGAATA 3´) into the hairpin. Both, human and mouse target sequences were validated previously [8]. For colocalization assays, the second expression cassette of pG-Shin2 vector, which encodes for EGFP, was modified by PCR insertion of a nuclear localization signal (PKKKRKV) at the N-terminus of the EGFP cDNA. (Forward primer: 5`ATCCACCGGTGCCGTTATGGGACCCAAGAAGAAGCGGAAAGTGAGCA AGGGCGAGGAG 3`; Reverse primer: 5’ GAGTCAGCTGAGCGAGGAAGC 3’). The PCR product containing NLS-EGFP was cloned into the AgeI and SalI sites of pG-Shin2. Targeting EGFP to the nucleus facilitated simultaneous detection of pG-Shin2-transfected cells and cytoplasmic proteins without interference.

Knockdown of NSF was performed using ON-TARGETplus siRNA duplexes designed to a previously validated target sequence [28], and obtained from Dharmacon. The siRNA duplexes were tested in a range of 10–100 nM and used at a final concentration of 10 nM for NSF. Cells maintained in Opti-MEM were transfected with the siRNA using Lipofectamine 2000 (Invitrogen) according to the manufacturer’s instructions, and processed after 48 h. As a control, the ON-TARGETplus non-targeting siRNA duplex #1 from Dharmacon was used.

### Cell Culture and DNA Transfection

HeLa cells and SW48 cells were acquired from the American Type Culture Collection (ATTC). PTP1B null (KO) cells and PTP1B reconstituted (WT) cells were kindly provided by B. Neel [29]. Cells were cultured in high-glucose Dulbecco´s Modified Eagle Medium (DMEM) containing L-glutamine and supplemented with 10% fetal bovine serum and 1% penicillin/streptomycin (Invitrogen) in a 5% CO_2_, 37°C incubator. Transient transfections were performed in 24-well tissue-culture plates (1–1.5 μg DNA/well), in 35 mm dishes (5 μg DNA/dish) or in 60 mm dishes (10 μg DNA/dish) using Lipofectamine 2000 (Invitrogen) for SW48 cells, or PEI (polyethylenimine) 87K (25 mM ethyleneimine monomer) [30] for HeLa cells. Transfections were performed according to the manufacturer's recommendations (LF: 2μl/μg DNA; PEI: 3μl/μg DNA). After 48 h of transfection, cells were harvested for analysis.

### Immunoprecipitation and Western Blotting

We followed previously described procedures [22]. Cells were lysed on ice (30 min) in a buffer containing 20 mM Tris-HCl, pH 7.4, 137 mM NaCl, 1% v/v Triton X-100, 1 mM PMSF, 10 μg/ml leupeptin, 5 μg/ml aprotinin and 10 mM NaF. Dishes were scrapped, and the cell suspension was centrifuged at 13.000 ***g*** for 10 min at 4°C. About 1 mg of supernatant protein was sequentially incubated at 4°C with 2 μg primary monoclonal antibodies (3 h), and protein G-Sepharose or protein A/G- magnetic beads (1.5 h). Immunocomplexes were washed three times with lysis buffer and boiled 5 min in SDS-PAGE sample buffer. Supernatants were fractionated by SDS-PAGE and transferred to polyvinyl difluoride (PVDF) or nitrocellulose membranes (Millipore). Blots were probed with primary antibodies, followed by HRP-conjugated secondary antibodies and revealed by enhanced chemiluminescence, or incubated with IR-680 fluorescent secondary antibodies and scanned with the Odyssey CLx infrared imaging system (LI-COR). Quantitative analysis of the signal intensity of the bands was performed using ImageJ software (Wayne Rasband, National Institutes of Health). For phosphotyrosine analysis, blots were stripped [22] and probed with 4G10 monoclonal antibody.

### Immunofluorescence and quantification methods

Cells were processed as described [22]. Briefly, cells attached to fibronectin-coated coverslips (20 μg/ml) were sequentially fixed with 4% paraformaldehyde in PBS for 20 min, permeabilized with 0.5% Triton X-100/PBS for 5 min, and blocked in 3% BSA/PBS for 1 h. Primary and secondary antibodies were incubated at room temperature for 1 h. Samples were mounted in Vectashield (Vector) and analyzed with a 60 x, 1.4 NA objective in a Nikon TE2000-U microscope (Nikon) coupled to an ORCA-AG cooled CCD camera (Hamamatsu) or an Olympus FV1000 confocal microscope (Olympus). To quantify signal overlapping, confocal optical sectioning of the entire cell volume was performed taking images (1024 x 1024 pixels, 16 bits) every 0.4 μm intervals on the zeta axis. Image stacks were processed with ImageJ. Background subtraction was as follows: For punctate features, a rolling ball algorithm (radius size 5 pixels) was applied to images. For reticular and large pleiomorphic membranous compartments, an extracellular region of interest (ROI) was subtracted to images. The fraction of HA puncta overlapping with ERGIC-53 puncta was determined in ROIs excluding the nuclear and perinuclear compartments. The fraction of HA puncta overlapping with the endoplasmic reticulum (ER) calnexin was analyzed in ROIs of peripheral areas showing well defined ER tubules. Images were segmented based on signal intensity (default algorithm). HA and ERGIC puncta in binary images were further filtered by size (0.07–0.5 μm^2^) and circularity (0.5–1). Binary images were multiplied by original images and then analyzed for colocalization using the JACOP plugin, using the default settings. Manders´ colocalization coefficients were obtained. To analyze HA and GalNacT colocalization, images from confocal stacks were thresholded to detect objects with intensities of one standard deviation above the mean. Under these conditions, the Golgi area, revealed by the GalNacT label, was included in the binary images. Signal overlapping in GalNacT and HA images was evaluated by calculating Manders coefficients.

### Statistical analysis

Statistical analyses were performed using GraphPad Prism version 5 (GraphPad Software). Two-tailed t-student and Mann-Whitney tests were used to compare two samples, and a one-way ANOVA, followed by the Dunnett´s multiple comparison post hoc test, was used for multiple comparisons. p < 0.05 was considered significant.

## Results

### Expression of p120 catenin is required for N-cadherin precursor processing

**Fig 1:**
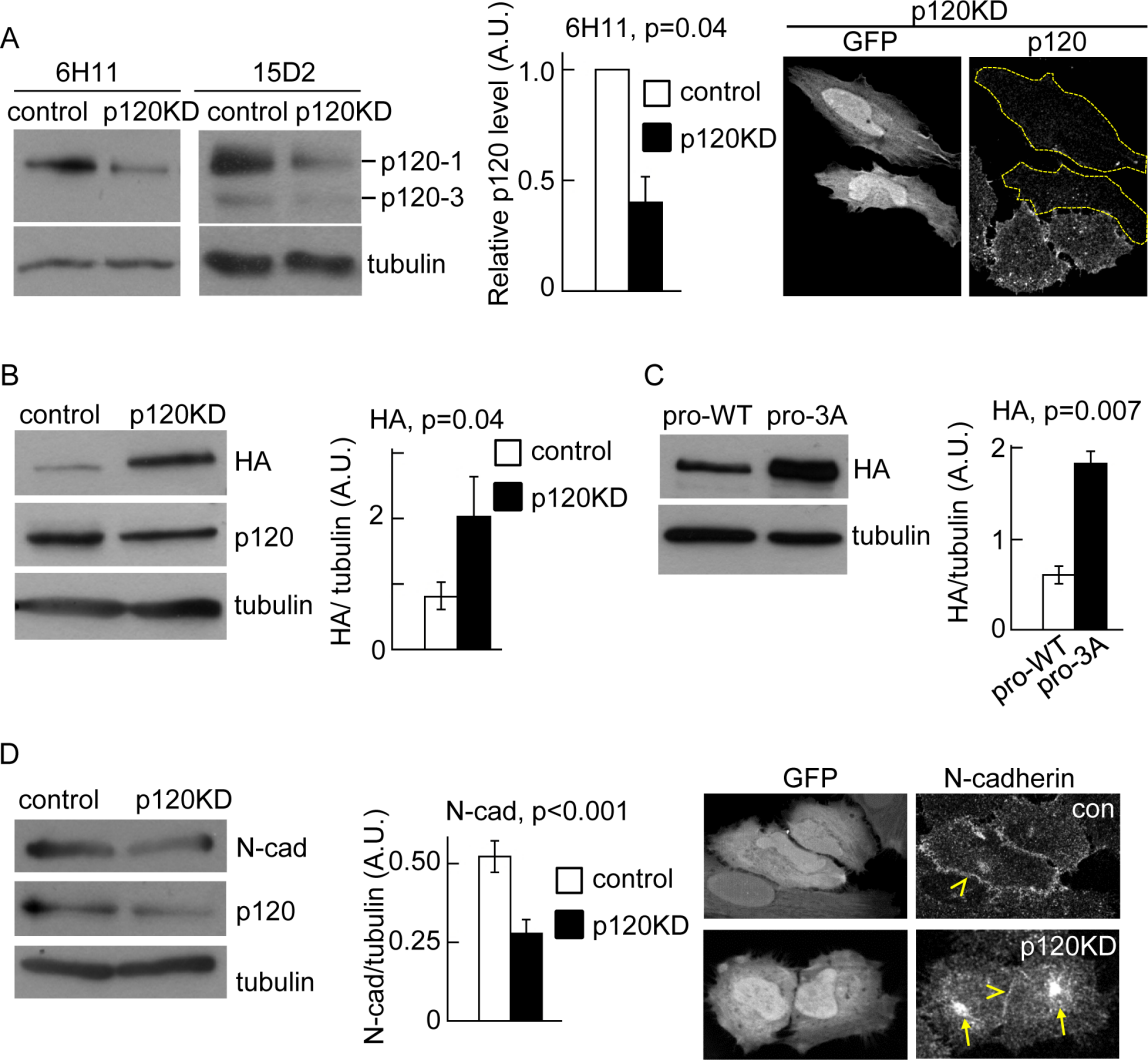
P120 catenin promotes N-cadherin precursor processing. (A) Left, Western blotting analysis of HeLa cells transfected with shRNAi targeting mouse p120 (control) or human p120 (p120KD). Specific antibodies were used for detection of endogenous levels of p120 isoforms and tubulin. Clone 15D2 recognizes the four isoforms of p120 and clone 6H11 recognizes isoforms 1 and 2. Both antibodies showed that p120-1 is the predominant isoform. The reduction of p120-1 in p120KD cells, relative to control is shown in the middle graph. Right panels are representative images of fluorescence microscopy showing the strong effect of p120KD in individual transfected cells (GFP-positive, dotted outlines). The p120 levels were detected by immunofluorescence with 6H11. (B) Western blotting of HeLa cells co-transfected with pro-WT and either, control or p120KD shRNAi. N-cadherin precursor levels, detected by HA labeling, were quantified and expressed as HA/tubulin ratios. (C) Western blotting of HeLa cells transfected with pro-WT or pro-3A. N-cadherin precursor levels, detected by HA labeling, were quantified and expressed as HA/tubulin ratios. (D) Western blotting showing endogenous N-cadherin levels in control and p120KD HeLa cells and immunofluorescence showing the distribution of endogenous N-cadherin in control and p120KD HeLa cells (identified by GFP expression, left panels). Yellow arrowheads at the right panels point intercellular junctions and yellow arrows point the intracellular accumulation of N-cadherin in p120KD cells. At least three repetitions were used for quantifications of blots. A. U. (arbitrary units). Bars in graphs represent means ± S.E.M. Statistical significance was determined by two-tailed Student´s *t*-test.

In a previous study we found that inefficient delivery of N-cadherin to the cell surface correlated with reduced association of p120 catenin to the N-cadherin precursor [22]. In addition, an N-cadherin mutant unable to bind p120 failed to traffic efficiently from ER to Golgi. Thus, we hypothesized that p120 catenin plays an active role promoting N-cadherin precursor trafficking at early stages of the biosynthetic pathway. To study the N-cadherin precursor trafficking in HeLa cells we used a previously characterized construct, HA-N-cadherin-GFP (pro-WT), which contains the hemagglutinin (HA) epitope inserted into the pro-peptide sequence at the N-terminus of N-cadherin, and EGFP at the C-terminus [22]. The relative levels and subcellular distribution of the precursor and the entire pool of N-cadherin can be determined by HA and EGFP detection, respectively. The pro-WT construct migrates slower than endogenous N-cadherin in SDS-PAGE and can be easily distinguished. Analysis by Western blotting probed with anti-N-cadherin, revealed that expression levels of pro-WT represented, on average, ~ 12% of the endogenous N-cadherin levels (Figure A in S1 File). Since in our experimental conditions about 40% of the total cell population is transfected, the expression of pro-WT in transfected cells must be ~ 30% of the endogenous N-cadherin. To investigate the role of p120 in pro-WT trafficking, we knocked down p120 expression in HeLa cells by expression of a small hairpin RNAi against a validated human p120 target sequence [8]. The shRNAi vector used for this purpose co-expresses EGFP as an independent cistron, allowing an easy identification of knockdown cells by microscopy [27]. As a control, we used a shRNAi vector encoding the target sequence of mouse p120, which differs by three bases from the human p120, and did not have any effect in human cells [8] (Figure B in S1 File). The levels of p120 were analyzed by Western blotting probed with two monoclonal antibodies: clone 6H11 recognizes an N-terminal epitope and detects isoforms 1 and 2, while clone 15D2 recognizes an epitope near the C-terminus of p120 and detects the four main p120 isoforms [31]. Consistent with previous work, we found that control HeLa cells express prominently the p120-1 isoform, and low levels of the p120-3 isoform (Fig 1A; [32]). In cells expressing the p120 shRNAi (p120KD), the levels of both p120 isoforms were reduced in a similar proportion. Quantification of p120-1 levels showed a reduction of 70% compared to the levels in cells expressing control shRNAi. The strong inhibition of p120 expression in individual EGFP positive cells was also confirmed by immunofluorescence microscopy (Fig 1A). In cells co-expressing the p120 shRNAi and pro-WT, the levels of N-cadherin precursor, revealed by anti-HA detection, increased ~2.4-fold compared to cells expressing control shRNAi (Fig 1B). Similar results were observed in HeLa cells expressing a construct containing a triple alanine substitution in the juxtamembrane domain which prevents p120 binding [5, 22]. This construct (pro-3A) also contained an HA epitope inserted within the pro-peptide to detect the precursor form [22]. Western blotting analysis using anti-HA revealed a ~3-fold increase in pro-3A levels compared to the control pro-WT construct (Fig 1C).

Previous studies have demonstrated that p120 levels act as a set point for determining cadherin levels [8, 9]. In agreement with this, we found that endogenous mature N-cadherin levels were significantly reduced in p120KD cells (Fig 1D). Immunofluorescence analysis further revealed a reduction of N-cadherin at intercellular junctions and an increase of the cytoplasmic pool (Fig 1D). Collectively, these results strongly suggest that p120 expression and binding to the N-cadherin precursor is required for trafficking from the ER, to the late compartment in the secretory pathway where it is proteolytically processed.

### P120 facilitates progression of the N-cadherin precursor through early stages of the secretory pathway

**Fig 2:**
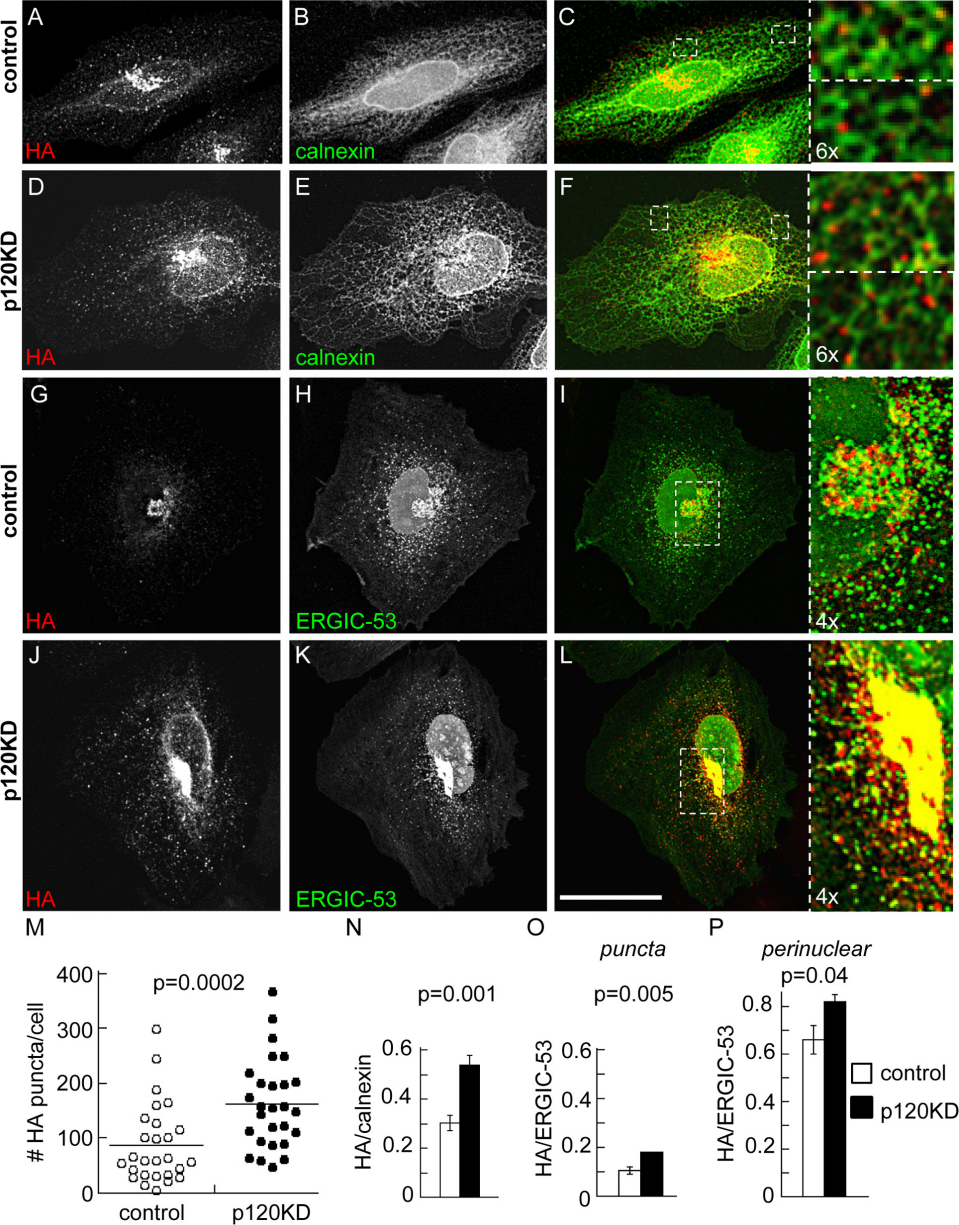
Distribution of N-cadherin precursor in p120KD HeLa cells. HeLa cells were co-transfected with pro-WT2 and either control (A-C, G-I) or p120KD (D-F, J-L) shRNAi. Cells were analyzed by confocal microscopy and representative images projected along the z-axis are shown. (A-F) Colocalization of N-cadherin precursor (HA, red label) with the ER marker calnexin (green label), detected by immunofluorescence. (G-L) Colocalization of the N-cadherin precursor (HA, red label) with transfected GFP-ERGIC-53 (green signal). Enlarged views from selected regions (white dotted boxes) are shown at the right. The expression of the control or p120KD shRNAi constructions in the analyzed cells is revealed by the modified nuclear-targeted GFP encoded by the vector (green channel). Scale bar in (L), 35 μm. (M) Number of HA puncta per cell in control and p120KD conditions (n = 27 cells per condition). Horizontal lanes indicate the position of means (control = 87,8; p120KD = 162.1). (N) Manders colocalization coefficients of the HA puncta overlapping calnexin in peripheral regions (control, n = 9 cells, total 422 puncta; p120KD, n = 7 cells, total 617 puncta). (O, P) Manders colocalization coefficients of the HA label overlapping GFP-ERGIC-53; independent analysis of the punctate (O) and clustered perinuclear distribution (P) of the HA label was performed (n = 24 cells per condition). Bars represent means ± S.E.M. Statistical significance was determined by a two-tailed Mann-Whitney test.

We predicted that accumulation of the N-cadherin precursor in conditions of p120 inhibition must occur in a compartment of the secretory pathway previous to the proteolytic removal of the pro-peptide at the TGN. To address this issue we performed colocalization analysis of the HA label with markers of ER-Golgi traffic compartments in stacks of optical sections obtained by confocal microscopy. For this study we used a variant of the pro-WT construct (called pro-WT2) in which the EGFP was eliminated from the N-cadherin C-terminus. In addition, the EGFP from the shRNAi vector was modified by addition of a nuclear localization sequence that delivers the protein to the nucleus and avoids the interference of EGFP in the colocalization analysis. The distribution of pro-WT2 in control cells was prominent in the perinuclear region (Fig 2A and 2G), where we previously showed an overlapping with Golgi markers [22]. In p120KD cells the N-cadherin precursor accumulated in the perinuclear compartment, but in addition, displayed a widespread punctate distribution throughout the cell (Fig 2D and 2J). Quantification of HA puncta per cell revealed a significant difference between p120KD and control cells (mean ± S.E.M., control: 88 ± 13; p120KD: 162 ± 15; Fig 2M). We examined whether the punctate distribution overlapped with the ER marker calnexin. High resolution confocal imaging revealed that pro-WT puncta partly overlapped with tubular ER in both, control and p120KD conditions (Fig 2C and 2F and insets). Quantification of Manders´ overlapping coefficients determined in peripheral regions of the ER network confirmed these observations and revealed higher colocalization of HA/calnexin in the p120KD condition (mean ± S.E.M., control: 0.30 ± 0.03, n = 422 HA puncta from 9 cells; p120KD: 0.54 ± 0.04, n = 617 puncta from 7 cells; Fig 2N). We then examined the colocalization of the precursor with ERGIC-53, a marker of the intermediate compartment between ER and Golgi, which distributes in punctate structures and in a perinuclear pleiomorphic compartment [33, 34]. Expression of GFP-ERGIC-53 in HeLa cells displayed the typical punctate and perinuclear distribution in both, control and p120KD conditions. Signal overlapping between HA and GFP-ERGIC-53 was not evident in the punctate distribution but was obvious in the perinuclear compartment (Fig 2I and 2L and insets). Quantification of the Manders´colocalization coefficients in punctate and perinuclear compartments confirmed these observations and revealed higher colocalization in the p120KD condition (Fig 2O and 2P). These results suggest a higher accumulation of the N-cadherin precursor at early traffic compartments under p120 inhibition.

### P120 regulates the recruitment of NSF to the N-cadherin precursor complex

We next examined potential mechanisms underlying the impaired N-cadherin precursor trafficking in the absence of p120. Upon considering several possibilities, we turned our attention to the N-ethylmaleimide sensitive factor (NSF). NSF is an ATPase essential for cis v/t-SNARE complex disassembly and maintenance of fusion competent pools of free v- and t-SNAREs [26]. It is therefore an important component of the vesicular trafficking machinery. NSF depletion by siRNA knockdown decreased the trans-epithelial electrical resistance of a cell monolayer [35]. Moreover, NSF is dephosphorylated and activated by the ER-resident protein tyrosine phosphatase PTP1B, promoting vesicle fusion events [28, 36–37]. We recently showed that PTP1B deficiency impaired ER to Golgi trafficking of the N-cadherin precursor, and this effect correlated with reduced p120 association to the precursor [22].

**Fig 3:**
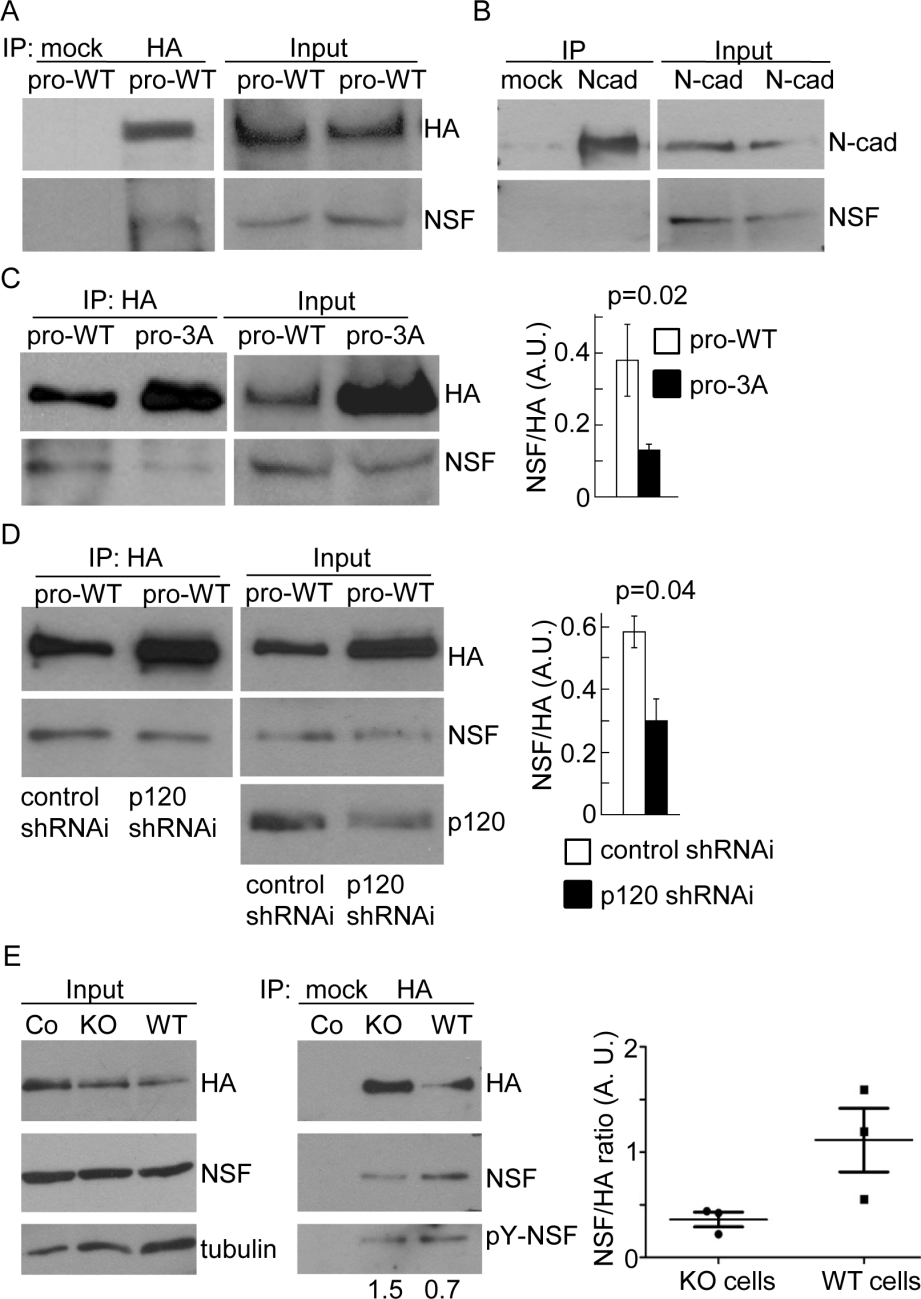
NSF association with the N-cadherin precursor. (A, B) Soluble protein extracts from HeLa cells transfected with pro-WT were sequentially immunoprecipitated with anti-HA and anti-N-cadherin, to isolate the precursor (A) and the mature (B) N-cadherin pools. Mock immunoprecipitations omitted the primary antibody. The presence of NSF in the complexes was analyzed in Western blotting probed with anti-NSF. (C) Immunoprecipitation of N-cadherin precursor from cells transfected with pro-WT or pro-3A constructs, and analysis of NSF in the complexes. (D) Cells co-transfected with pro-WT and either control or p120shRNAi. N-cadherin precursor was isolated by HA-immunoprecipitation and the presence of NSF in the complex was detected in blots. The graphs in (C) and (D) represent quantifications of NSF bands, normalized to the HA signal. (E) Murine PTP1B KO and WT cell lines transfected with pro-WT were immunoprecipitated with anti-HA, and the protein complexes were analyzed by Western blotting probed with antibodies against HA, NSF, phosphotyrosine and tubulin. Numbers under the panels indicate the pY-NSF/NSF ratio. The graph at the right shows the quantification of NSF bands normalized to HA. In all cases, at least three independent experiments were used for quantifications. Data represent means ± S.E.M. Statistical significance was determined by two-tailed Student´s *t*-test.

Thus, we sought to examine whether NSF is present in the complex of proteins associated with the N-cadherin precursor. We transfected HeLa cells with pro-WT and isolated the precursor complex by HA immunoprecipitation. Western blotting probed with different monoclonal antibodies against NSF consistently detected a band of ~76 kDa in the complex, which is not seen in the mock immunoprecipitation (Fig 3A). Remarkably, subsequent immunoprecipitation to isolate mature N-cadherin did not show associated NSF (Fig 3B). To determine if p120 was involved in NSF association to the N-cadherin precursor, we analyzed the presence of NSF in HA immunoprecipitates of cells transfected with pro-WT and the mutant pro-3A which cannot bind p120. As it is shown in Fig 1C, pro-3A precursor accumulated to a higher level than pro-WT; however, the amount of NSF associated with pro-3A precursor was significantly reduced compared to pro-WT (Fig 3C). Quantification of data revealed that NSF/HA ratios in pro-3A expressing cells were a third of those in pro-WT expressing cells. These results were confirmed in p120KD HeLa cells transfected with pro-WT (Fig 3D). Our data suggest that association of p120 to the cytoplasmic domain of N-cadherin precursor contributes to the recruitment of NSF to the complex. Since association of p120 with the N-cadherin precursor is impaired under PTP1B deficiency [22], we predicted that the amount of NSF co-immunoprecipitated with the N-cadherin precursor in murine PTP1B knockout (KO) cells should be impaired compared to KO cells reconstituted with PTP1B (WT). Results from three independent experiments revealed that NSF/HA ratios were consistently higher in WT cells than in KO cells (Fig 3E). Stripping the membranes and re-probing them with anti-phosphotyrosine revealed that NSF was phosphorylated in both, KO and WT cells, although the ratio pY/NSF in KO cells was higher than in WT cells in two experiments.

### NSF function is required for N-cadherin trafficking and processing

**Fig 4:**
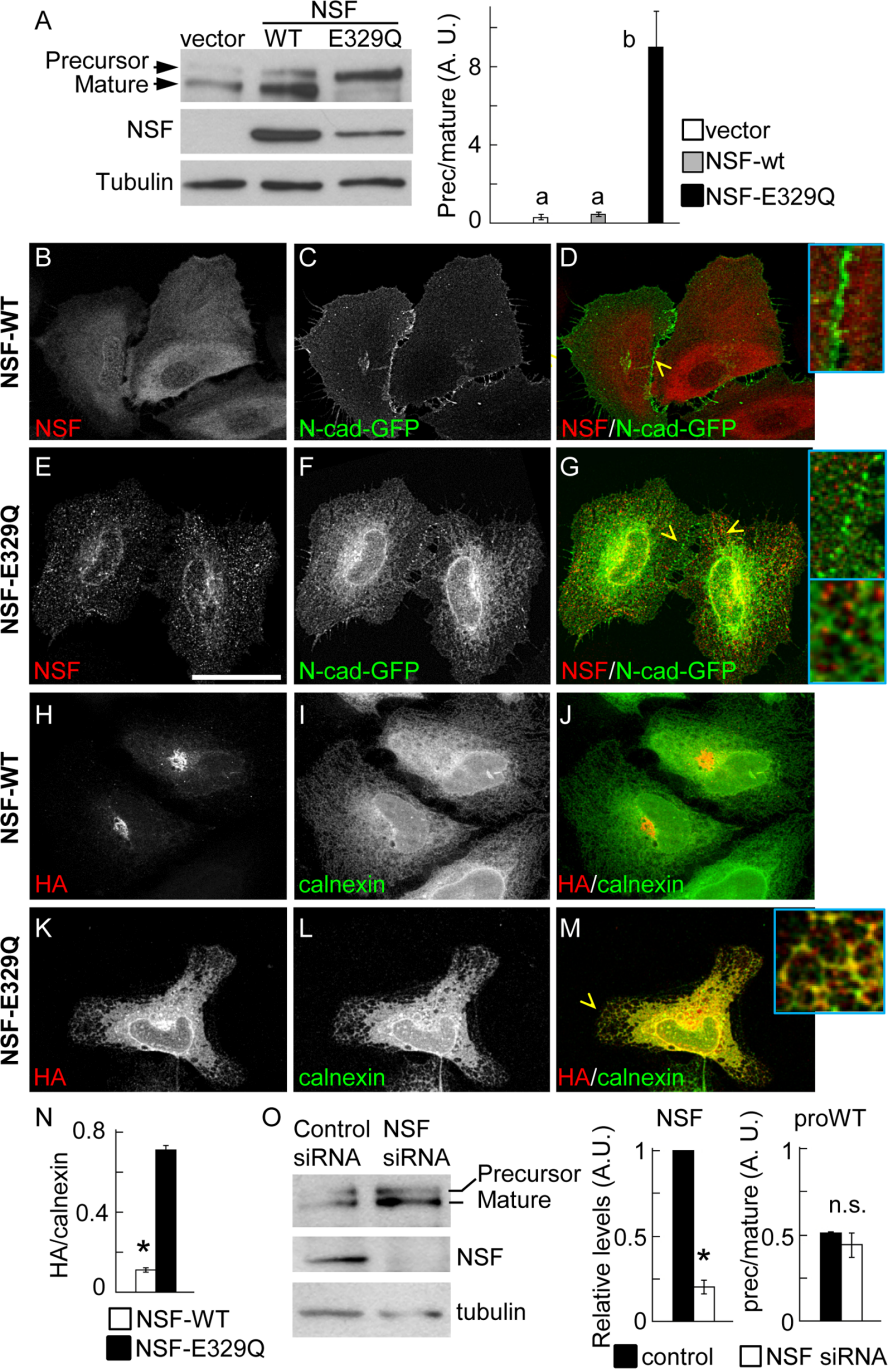
Function of NSF in N-cadherin trafficking. (A) Western blotting analysis of HeLa cells co-transfected with pro-WT, and empty vector or vector encoding NSF-WT or NSF-E329Q. Precursor and mature forms of pro-WT were detected with anti-N-cadherin. The lower size of the endogenous N-cadherin is not depicted. Visualization of ectopic NSF-WT and NSF-E329Q signals under non-saturating conditions did not allow detection of endogenous NSF (first lane). At least three independent experiments were used for quantification of precursor and mature forms of pro-WT (right graph). Bars represent means ± S.E.M. of precursor/mature ratios normalized to tubulin. Statistical significance (p < 0.05) was determined using one-way ANOVA, followed by the Dunnett´s multiple comparison *post hoc* test. Different letters indicate statistically different means. (B-G) Distribution of N-cadherin-GFP and ectopic NSF constructs. HeLa cells were co-transfected with N-cadherin-GFP and either NSF-WT (B-D) or NSF-E329Q (E-G). (H-M) Colocalization of N-cadherin precursor (HA, red label) and the ER marker calnexin (green label) in cells co-transfected with pro-WT2 and NSF-WT (H-J) or NSF-E329Q (K-M). The expression of exogenous NSF constructs was verified in triple label samples (not shown). (N) Manders colocalization coefficients of the HA/calnexin signal overlapping in NSF-WT (n = 15 cells) and NSF-E329Q (n = 23 cells) conditions. Bars represent means ± S.E.M. Statistical significance was determined by a two-tailed Mann-Whitney test. Cells were analyzed by confocal microscopy and representative images projected along the z-axis are shown. Enlarged views (4x) from selected regions (yellow arrowheads) are shown as insets. Scale bar in (E), 35 μm. (O) HeLa cells were co-transfected with proWT and non-targeting siRNA duplexes (Control) or NSF siRNA duplexes (NSF siRNA). After 48 h, precursor and mature forms of the proWT construct, NSF and tubulin were analyzed by Western blotting. Quantification of NSF levels (NSF siRNA values relativized to control siRNA) and precursor/mature ratios in control and NSF siRNA conditions is shown at the right. Bars represent means ± S.E.M. from three independent experiments. Statistical significance (asterisks, p < 0.05) was determined by a two-tailed Student´s *t*-test.

To determine the role of NSF in N-cadherin precursor trafficking and processing, HeLa cells were co-transfected with pro-WT, and either NSF-WT or NSF-E329Q constructs. NSF-E329Q is deficient in ATP hydrolysis and therefore inhibits the function of endogenous NSF, resulting in a dominant negative effect [38]. Western blotting probed with anti-N-cadherin, to simultaneously detect the ectopic precursor and mature N-cadherin forms, revealed that in cells expressing the empty vector or overexpressing NSF-WT, more than 70% of the ectopic N-cadherin is processed to the mature form, while in cells expressing NSF-E329Q more than 90% of the ectopic N-cadherin accumulated as a precursor form (Fig 4A). To complement these studies we analyzed, by confocal microscopy, the subcellular distribution of precursor and mature forms of N-cadherin in HeLa cells expressing NSF-WT or NSF-E329Q. The distribution of over-expressed NSF-WT was diffuse in the cytosol, while that of NSF-E329Q followed a punctate pattern throughout the cell, in agreement with previous findings [39] (Fig 4B and 4E). The distribution of N-cadherin-GFP in cells expressing NSF-WT was prominent at intercellular junctions (Fig 4C and 4D, inset). In contrast, the distribution of N-cadherin-GFP in cells expressing NSF-E329Q showed a discontinuous pattern at intercellular junctions and a significant accumulation at the ER, frequently overlapping with NSF-E329Q puncta (Fig 4F and 4G, inset). The distribution of the N-cadherin precursor was evaluated in cells transfected with pro-WT2. As expected, the N-cadherin precursor in cells expressing NSF-WT was visualized at the perinuclear region, in a Golgi location, and showed minimal colocalization with calnexin (Fig 4H, 4J and 4N). In contrast, in cells expressing NSF-E329Q most of N-cadherin precursor was retained at the ER, where it showed a tight colocalization with calnexin (Fig 4K and 4M, inset, 4N). Strikingly, siRNA-mediated knockdown of NSF did not affect the rate of N-cadherin precursor processing (Fig 4O).

### P120 reconstitution in p120-defficient cells rescues N-cadherin trafficking and processing

**Fig 5:**
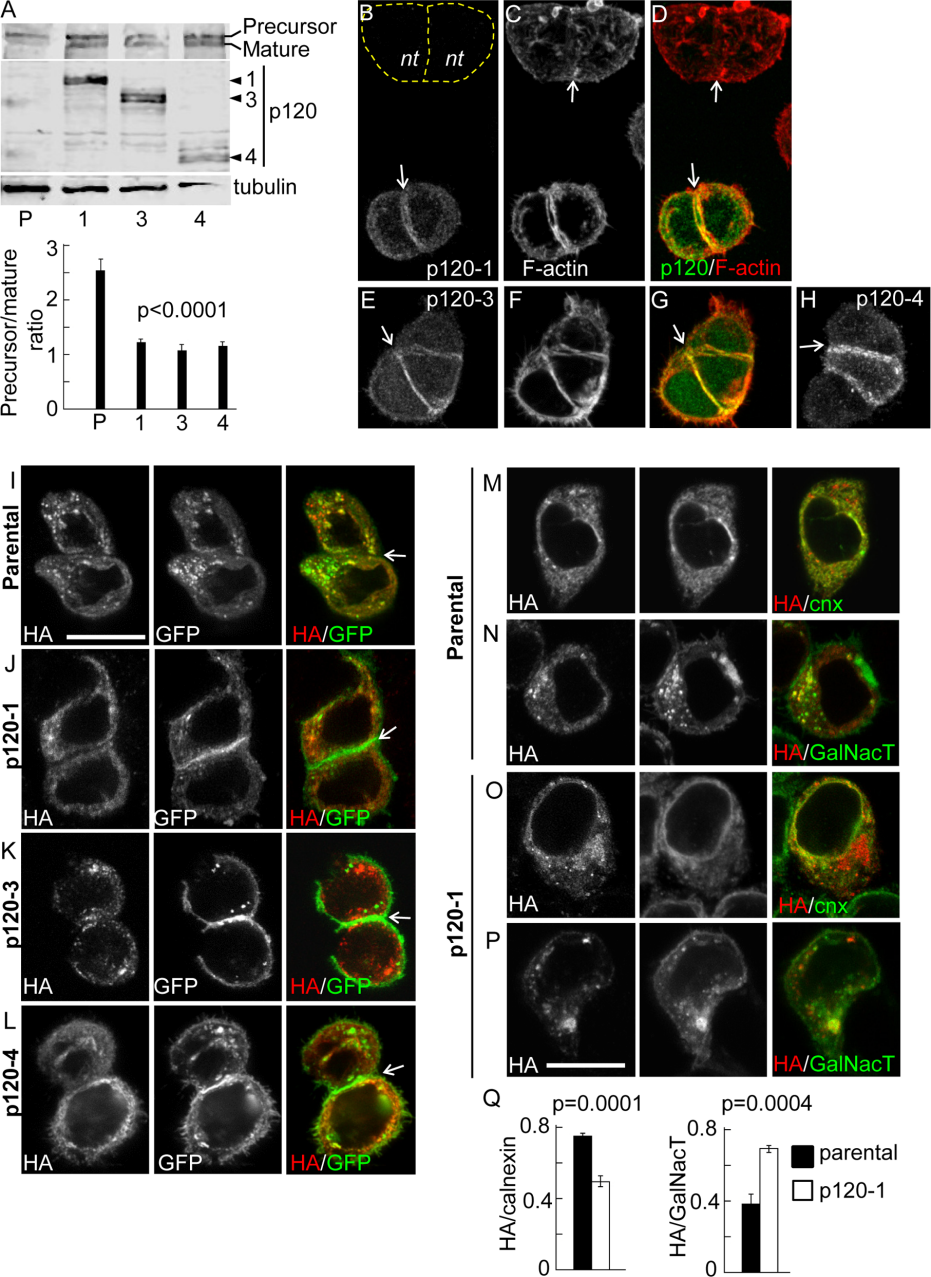
N-cadherin distribution in SW48 colon carcinoma cells reconstituted with p120. (A) Cells were co-transfected with pro-WT and p120 isoforms 1, 3 or 4. Soluble protein extracts from parental (P) and reconstituted cells (1, 3 and 4) were analyzed in Western blotting probed with anti-N-cadherin to recognize the precursor and mature forms of pro-WT, the p120 isoforms (clone 15D2), and tubulin. The graph shows quantification of precursor/mature ratios normalized to tubulin. (B-H) Confocal analysis of representative cells reconstituted with isoform 1 (B-D), 3 (E-G) and 4 (H). Cells were labeled for p120 (B, E, H) and F-actin (C, F). Arrows point intercellular junctions. "*nt*" in (B) indicates non-transfected cells. (I-L) Distribution of precursor and mature forms of N-cadherin in parental cells expressing pro-WT and empty vector (I), or vectors encoding p120-1 (J), p120-3 (K) or p120-4 (L). The distribution of ectopic N-cadherin precursor (red label) and total N-cadherin (green label) was detected by anti-HA and GFP fluorescence, respectively. (M-P) Colocalization of the N-cadherin precursor with ER and Golgi markers. Cells expressing pro-WT2 in parental (M) and reconstituted with p120-1 (O) conditions were double-labeled for HA (red signal) and calnexin (green signal). The expression of p120-1 construct was verified in triple label samples (not shown). Note the redistribution of the HA signal to the perinuclear compartment in p120-1 reconstituted cells. The Golgi apparatus was revealed by GalNacT-DsRed transfection in parental (N) and p120-1 reconstituted (P) cells. Note the tight overlapping of the HA label (shown in red) with GalNacT-DsRed (shown in green) in the Golgi. (Q) Manders colocalization coefficients calculated from HA and calnexin (n = 13 cells), and HA and GalNacT (n = 13 cells) signals. Bars represent means ± S.E.M. Statistical significance was determined by a two-tailed Mann-Whitney test. All cell images represent equatorial confocal planes and white arrows point intercellular junctions. Scale bar in (P), 14 μm.

Most cell types express multiple isoforms of p120, generated by alternative splicing of a single gene [40]. The use of different translation start codons results in four isoforms, p120-1, 2, 3, and 4. All isoforms contain a central armadillo domain, which mediates binding to cadherin, but differ in the length of the N-terminal regulatory domain, being p120-1 the longest isoform, and p120-2, -3 and -4 progressively shorter at the N-terminal region. The p120 gene in the SW48 human colon carcinoma cell line contains a nonsense mutation in exon 7, yielding a premature stop codon and negligible expression levels of the protein [41]. We used SW48 cells to test if different p120 isoforms were capable of promoting N-cadherin trafficking and processing. Western blotting analysis of SW48 cells transfected with pro-WT and probed with anti-N-cadherin, to simultaneously detect the precursor and mature N-cadherin species revealed that the precursor was the predominant form (Fig 5A). Endogenous levels of N-cadherin were negligible (not shown). Using an antibody that recognizes all p120 isoforms (clone 15D2, [31]) we also confirmed the negligible expression of endogenous p120 isoforms (Fig 5A). Individual reconstitution of SW48 cells with p120-1, -3, and -4 promoted the processing of the N-cadherin precursor and the appearance of mature N-cadherin, visualized as a lower molecular weight band in blots (Fig 5A). Confocal analysis of cells at equatorial planes revealed that all p120 isoforms transfected in SW48 cells accumulated at intercellular junctions, as expected (Fig 5B–5H). Parental, non transfected cells did not develop conspicuous intercellular junctions, as revealed by F-actin labeling (Fig 5B–5D, "*nt*"). In addition, expression of pro-WT in parental SW48 cells, did not show accumulation of GFP at intercellular junctions, both the HA (precursor cadherin) and GFP label (total N-cadherin) colocalized at intracellular compartments (Fig 5I). In contrast, cells reconstituted with different p120 isoforms showed the expected intracellular HA (and GFP) label, but a prominent and exclusive GFP signal accumulated at intercellular junctions, consistent with the production of mature and functional N-cadherin (Fig 5J–5L). In addition, p120-reconstituted cells tend to form more compacted cellular aggregates than parental cells, in agreement with previous findings showing that expression of p120 in SW48 cells induced a significant compaction of epithelial colonies [41]. We confirmed the expression of p120 in reconstituted cells in separate experiments (not shown). Confocal colocalization analysis of the pro-WT2 construct (lacking the GFP at the C-terminus) revealed that in parental SW48 cells the N-cadherin precursor (HA label) colocalized with calnexin (ER marker) and in a minor degree with GalNacT (Golgi marker, [42] (Fig 5M, 5N and 5Q). In contrast, in cells reconstituted with p120 isoforms 1, 3 and 4, the colocalization of N-cadherin precursor with calnexin was less evident, while its colocalization with GalNacT increased (Fig 5O, 5P and 5Q; only data of isoform 1 is shown). Taken together, the reconstitution of different p120 isoforms in SW48 cells confirm the results of p120 knockdown in HeLa cells, strongly indicating that p120 expression promotes the trafficking and processing of the N-cadherin precursor and the surface localization of mature N-cadherin. This function of p120 can be performed by all isoforms tested.

**Fig 6:**
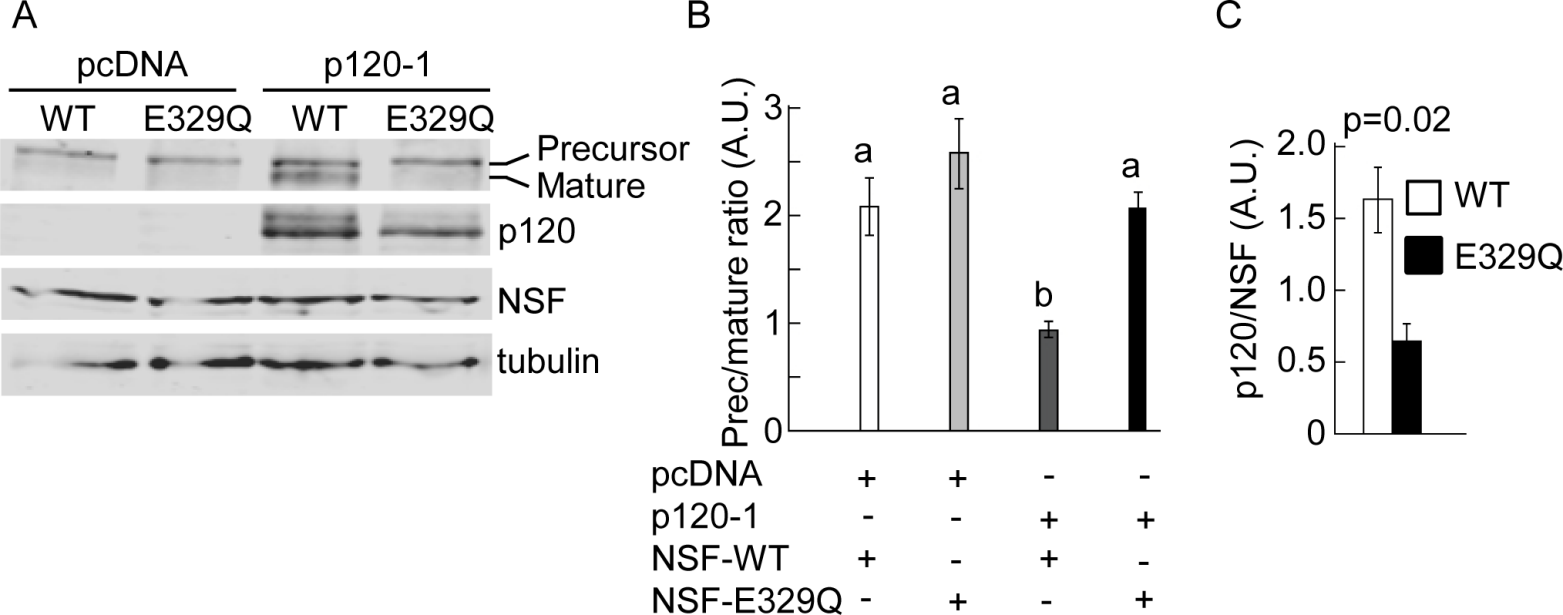
Combinatorial analysis of p120 and NSF in N-cadherin processing. (A) SW48 cells expressing pro-WT in combination with empty vector (pcDNA) or p120-1, and NSF-WT (WT) or NSF-E329Q (E329Q). Shown is a representative blot probed with anti-N-cadherin to detect the precursor and mature form of N-cadherin, anti-p120, anti-NSF, and anti-tubulin. (B, C) Quantification of N-cadherin precursor/ mature ratio (B) or p120/ NSF ratio (C) from blot scans expressed in arbitrary units (A. U.). Graph bars represent means ± S.E.M. from three independent experiments. Statistical significance (p < 0.05) was determined using one-way ANOVA, followed by the Dunnett´s multiple comparison *post hoc* test (B), or a two-tailed Student´s *t*-test (C). Different letters in (B) indicate statistically different means.

To further confirm the role of NSF and p120 in N-cadherin precursor processing, SW48 cells were co-transfected with pro-WT along with empty vector (pcDNA) or pcDNA-p120-1 in combination with either NSF-WT or NSF-E329Q. Western blotting analysis revealed that overexpression of functional NSF in parental cells was not enough to promote N-cadherin processing in absence of p120 expression (Fig 6A). Also, reconstitution of parental cells with p120 isoform 1 was not enough to promote the processing of N-cadherin if the dominant negative NSF-E329Q was co-expressed. Expression of both, NSF-WT and p120-1 was necessary for N-cadherin precursor processing to the mature form (Fig 6A and 6B). In three independent repetitions of this experiment we consistently noticed lower levels of p120 in cells expressing NSF-E329Q, compared to those expressing NSF-WT. Quantification of p120 and NSF bands in densitometric scans, and comparison of p120/NSF ratios (or p120/tubulin ratios, not shown), confirmed the statistical significance of these observations (NSF-WT mean: 1.6 ± 0.23 vs NSF-E329Q mean: 0,65 ± 0.12) (Fig 6C).

## Discussion

After biosynthesis at the ER, cadherins are delivered to the cell surface where they mediate homophilic, Ca^+2^-dependent intercellular adhesion. Adhesion strength is modulated by the amount of cadherin at the cell surface, which results from a complex interplay among biosynthesis, degradation, traffic, and anchorage to the cytoskeleton [3, 43–45]. P120 and β-catenin are major direct interacting partners of cadherins. They bind to different sites in the cytoplasmic domain of cadherin precursors soon after their insertion at the ER membranes, trafficking as a complex towards the cell surface [17–19, 46, 47].

The present study proposes that early anterograde traffic of nascent cadherin is regulated by associated p120 catenin, suggesting a mechanism by which p120 catenin recruits NSF to the cadherin precursor complex, facilitating its movement from the ER to Golgi. So far it was well established that p120 association to mature cadherin at the cell surface prevented its internalization and degradation, acting as a "set-point" for cadherin expression [48]. However, the role of p120 in cadherin traffic through the biosynthetic pathway is intriguing and less understood. Pulse-chase experiments combined with surface biotinylation performed in human cervical carcinoma A431 cells, revealed that p120 knockdown (p120KD) did not affect E-cadherin biosynthesis and traffic to the cell surface [8]. However, other studies showed that p120 associated directly with microtubules and kinesin, and disruption of p120/kinesin interaction impaired the delivery of nascent N-cadherin to newly forming junctions [21, 49–51]. It should be noted that these studies suggest a role of p120 in post-Golgi delivery of N-cadherin towards the plasma membrane. Recent work from our laboratory, however, suggested that p120 could also be implicated in ER to Golgi transport of the N-cadherin precursor [22]. We found that in fibroblasts deficient in protein tyrosine phosphatase 1B (PTP1B) expression, the passage of the N-cadherin precursor from ER to Golgi was impaired. This defect correlated with decreased association of p120 to the precursor. Additionally, expression of HA-N-cadherin-3A mutant unable to bind p120, was more sensitive to endoglycosidase H than wild type N-cadherin, suggesting a defect in transport of the precursor from ER to Golgi [22]. These results, however, do not rule out the possibility that other partners, which bind to overlapping sites within the juxtamembrane region, such as presenilin-1, ankyrin-G and GRIP-1 could also be implicated [23–25]. Thus, in the present study we specifically inhibited p120 expression in HeLa cells by shRNAi, and provided a direct evidence of a p120 function in the ER-Golgi traffic of N-cadherin precursor. High resolution confocal analysis of p120KD cells revealed a punctate distribution of the N-cadherin precursor, which significantly overlapped with ER marker calnexin. In control conditions, however, most of the N-cadherin precursor accumulated at the Golgi apparatus and puncta distribution is rare. Additionally, in p120KD cells the N-cadherin precursor colocalized more tightly with the intermediate compartment marker ERGIC-53 than in control conditions. These results suggest that, under p120 inhibition, the N-cadherin precursor is able to aggregate at the ER, but its export and transport through the intermediate compartment is impaired. In normal conditions, and following arrival of the cadherin precursor to the *trans*-Golgi network (TGN), the propeptide is cleaved by furin proteases [15–17]. Consistent with an impaired flow of N-cadherin precursor from the ER to TGN in p120KD cells, we found a significant accumulation of precursor levels compared to control conditions. These observations were confirmed in HeLa cells transfected with the N-cadherin mutant unable to bind p120. In addition, the reconstitution of p120 expression in the p120-defficient SW48 cell line, promoted N-cadherin traffic and processing. We found that isoforms p120-1, -3 and -4 have an equivalent function. These isoforms share the conserved cadherin-binding domain and the C-terminal region, but differ in the length and presence of regulatory motifs localized in the N-terminal region [40]. Isoform-1 is the longest protein and contains the full length N-terminal domain, bearing a putative coiled-coil motif and a phosphorylatable regulatory domain. Isoform-3 lacks the coiled-coil motif but retains the regulatory domain, and isoform-4 lacks the entire N-terminal domain (~300 amino acids). Our results suggest that the capacity to promote trafficking and processing of the N-cadherin precursor does not reside in the N-terminal region of p120. However, we cannot rule out a modulatory effect of this region, as it has been reported for p120 function controlling cadherin dynamics [12].

Depletion of p120 could alter the anterograde traffic of the N-cadherin precursor through an indirect effect, for example impairing the association of β-catenin. Indeed, previous work in MDCK cells showed that expression of E-cadherin mutants with deletions or with inactivating amino acid substitutions in the β-catenin binding region, were mostly retained at the ER [47], or accumulated in Golgi and post-Golgi compartments [52]. It is not clear, however, if these mutants still associate efficiently with p120. Beta catenin could facilitate the export of N-cadherin from ER through binding to a PX-RICS-14-3-3ζ/θ-dynein-dynactin complex, since RNAi depletion of these components lead to N-cadherin retention at the ER [53]. Moreover, recent structural analysis revealed a novel functional consequence of β-catenin association to cadherin [54]. Beta catenin could contribute to strengthen cadherin-p120 association by recruiting α-catenin to the complex, and promoting additional contacts between p120 and α-catenin. However, most short term experiments inhibiting p120 expression or association with cadherin did not show a correlative effect on β-catenin. It was noted previously that p120 knockdown in SK-CO15 colon carcinoma cells did not affect β-catenin expression levels [35]. Similarly, we did not observe reduction of β-catenin levels by p120 knockdown in HeLa cells (results not shown). In addition, we and others clearly showed that β-catenin association with N-cadherin precursor was not coupled to p120 dissociation [7, 22, 55]. Thus, our present data on N-cadherin precursor traffic likely reflect the direct consequence of loosing p120 function.

What is the mechanism by which p120 associated with the N-cadherin precursor promotes ER to Golgi traffic? A major finding of the present work is that NSF is recruited to the N-cadherin precursor complex in a p120- and PTP1B-dependent manner. Previously we found that binding of p120 to the N-cadherin precursor, and ER to Golgi traffic, was impaired in PTP1B deficient cells [22]. Together, these results suggest a functional interplay among PTP1B, p120 and NSF, with consequences in N-cadherin traffic. PTP1B localization at the cytosolic face of ER membranes [56] may facilitate dephosphorylation of proteins associated with the cytoplasmic domain of the N-cadherin precursor, such as p120 and beta catenin [17, 20, 22]. Besides promoting the association of p120 with the N-cadherin precursor, and indirectly the recruitment of NSF to the complex, PTP1B may also have a direct effect on NSF activity. Indeed, three laboratories have independently shown a positive effect of PTP1B in membrane fusion, likely by dephosphorylation and activation of NSF [28, 36, 37]. Our results showing higher retention of phosphotyrosine in the NSF fraction associated with the N-cadherin precursor in KO cells compared to WT cells suggest this possibility.

As a general regulator of membrane fusion, NSF is likely required not only for ER-Golgi traffic but also for post-Golgi events. However, we were unable to detect NSF in complexes of mature N-cadherin, suggesting that different mechanisms regulate the composition of N-cadherin complexes at specific subcellular locations and functional stages. Regulation of protein trafficking to the plasma membrane in complexes with NSF has recently been described for transmembrane receptors, including the α-amino-3-hydroxy-5-methyl-4-isoxazolepropionic acid (AMPA) subtype of glutamate receptors [26]. These complexes could be linked with cadherins by multi-PDZ domain scaffold proteins such as the glutamate receptor interacting protein (GRIP), which interacts with the p120 relative δ-catenin [57]. GRIP also links AMPA receptors and N-cadherin with the anterograde motor protein KIF5C, ensuring the proper co-deliver of N-cadherin and AMPA receptors to dendritic spines [25]. Interestingly, δ-catenin binds to GRIP through a C-terminus motif. This is relevant to our finding that all isoforms of p120 tested have equivalent capacity to promote N-cadherin trafficking, suggesting that the molecular determinant implicated in NSF recruitment must reside in the armadillo and/or the C-terminal domain. Both mechanisms, the association of N-cadherin to KIF5C through GRIP and the association of NSF to the N-cadherin precursor (described in this work), could work together to ensure the proper delivery of N-cadherin to the cell surface.

**Fig 7:**
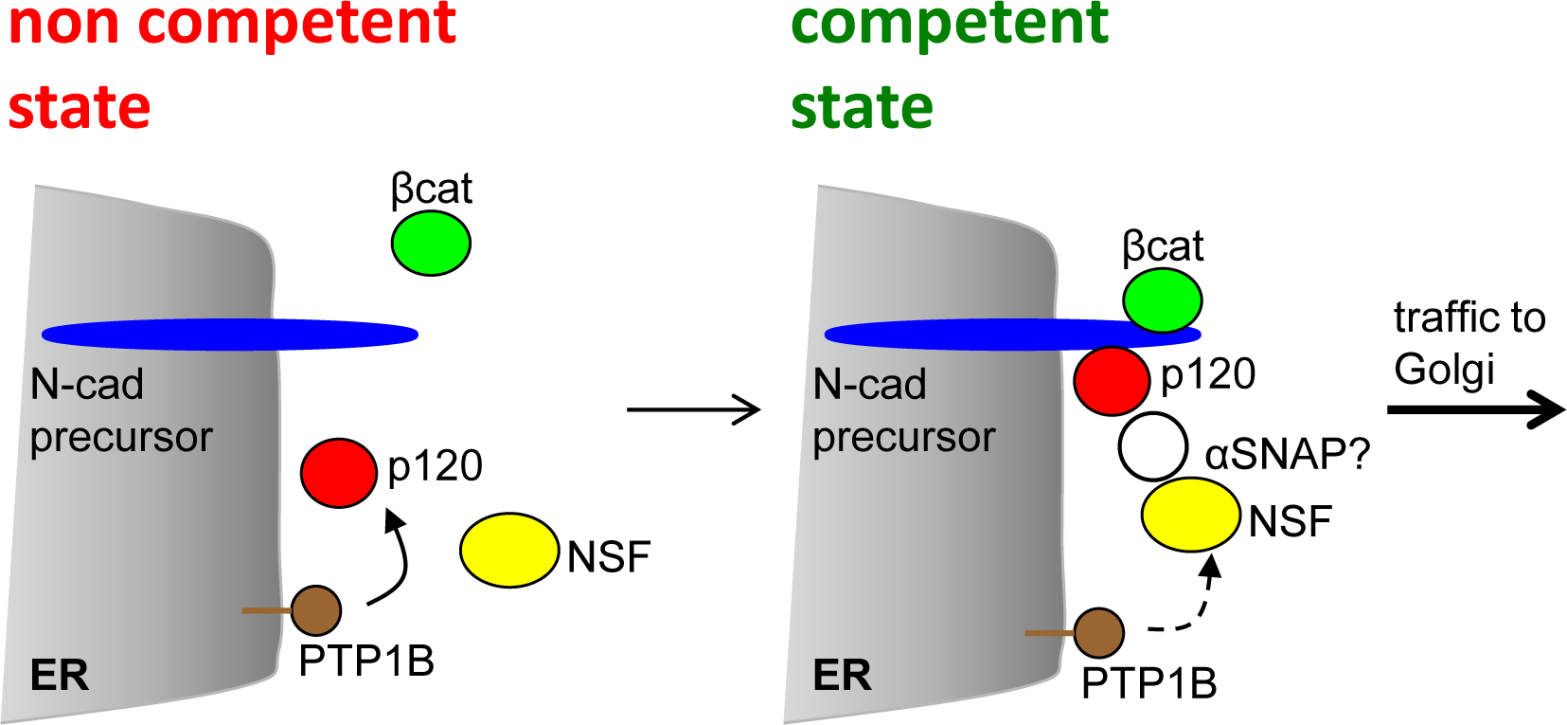
Model representing the complex assembly required for N-cadherin precursor traffic at the ER. N-cadherin precursor (blue) is co-translationally inserted at the ER membrane, with the C-terminus localized in the cytoplasm. Binding of p120 (red) and β-catenin (green) to the precursor cytoplasmic domain occurs at the ER. PTP1B (brown) localized at the ER dephosphorylates p120 and stabilize its association with the complex [22]. Perturbation of these events impairs traffic. In a traffic competent state, p120 recruits NSF (yellow) to the complex, where is likely dephosphorylated and activated by PTP1B. The recruitment of αSNAP (white) to the N-cadherin precursor complex, and its role in ER-Golgi traffic, remains to be confirmed.

Our results showed that impairing NSF function with the dominant negative construct NSF-E329Q led to a dramatic retention of N-cadherin precursor at the ER, impairing its processing to the mature form, and leading to a significant reduction of N-cadherin at intercellular junctions. Unexpectedly, NSF depletion by siRNA did not affect the processing of the N-cadherin precursor. Although we cannot completely exclude that a residual fraction of NSF may still fulfill the requirements for the N-cadherin precursor traffic, these results suggest a non essential role of NSF in the ER-Golgi movement of the N-cadherin precursor. In agreement with this, recent studies in NSF-depleted SK-CO15 colonic epithelial cells showed a lack of effect on the distributions of KDEL-R and GM130 [58]. However, depletion of the NSF homolog, p97, caused a redistribution of these markers from a well defined Golgi localization in control cells to a punctate distribution scattered throughout the cell, suggesting an impairment of ER-Golgi traffic [58]. Thus, the lack of effect of NSF siRNA depletion in our experiments may reflect compensatory functions performed by p97. The strong effect of the dominant negative NSF construct, observed in the present work, may reflect a "substrate trap" activity, likely sequestering the adaptor protein αSNAP [39, 58]. Recent work showed that RNAi depletion of αSNAP in SK-CO15 cells impaired epithelial junctions, and downregulated p120 catenin and E-cadherin, but not β-catenin expression [35]. Strikingly, αSNAP knockdown also impaired ER-Golgi transport, and produced accumulation of ER-glycosylated forms of the β1 integrin precursor, effects that are not mimicked by NSF depletion [35, 58]. The mechanistic aspects of αSNAP function in ER-Golgi traffic are still unknown, although experimental evidence suggests a role in pre-docking/docking steps during the membrane fusion process [59]. Our results expressing the dominant negative NSF construct in HeLa and SW48 cells recreate several effects of the αSNAP knockdown, including the impairment of intercellular junctions and ER-Golgi transport, as well as the downregulation of p120 levels [35, 58]. Thus, we cannot rule out that the dominant negative NSF-E329Q effects on N-cadherin traffic may be related to indirect inhibition of αSNAP. It remains to be determined if αSNAP, which recruits NSF to membranes, is present in the complex of proteins associated with the N-cadherin precursor, and the potential p120 dependency. We envisage a scenario in which ER-bound PTP1B function ensures p120-dependent recruitment of NSF to the N-cadherin precursor complexes. NSF activation, likely by PTP1B, facilitates membrane fusion events associated with vesicular traffic initiated at the ER (Fig 7). In the absence of NSF, its homolog p97 likely compensates NSF function.

## Supporting Information

S1 File Figure A: Expression levels of pro-WT. The HA-N-cadherin-GFP (pro-WT) construct was transfected in HeLa cells and the expression levels were analyzed by Western blotting with anti-N-cadherin, to allow for the direct comparison with endogenous N-cadherin levels. Due to the GFP fusion, pro-WT migrates slower than endogenous N-cadherin. Under our detection conditions essentially all ectopic and endogenous N-cadherin correspond to mature, processed proteins. Representative blots of two independent experiments are shown at the left. Quantification of the bands is shown in the graph. Bars represent means ± S.E.M. from five experiments. Figure B: Effect of p120 shRNAi targeting. (A) Western blotting analysis of HeLa cells transfected with empty pG-Shin2 vector (ve), the vector encoding the human p120 targeting sequence (hu), or the equivalent mouse p120 targeting sequence (mo). Blots were probed with anti-p120 (clone 6H11), anti-N-cadherin, and anti-tubulin. Only the human p120 targeting sequence showed a marked effect on p120 expression. A representative blot of 3 independent experiments is shown. (B-D) HeLa cells expressing the control, mouse p120 targeting sequence. Immunofluorescence with anti-p120 reveals similar p120 levels (red signal) in transfected (GFP positive cells) and non transfected cells. Scale bar in (D), 35 μm.
(PDF)

## References

[1] Berx G, Van Roy F (2009) Involvement of members of the cadherin superfamily in cancer. Cold Spring Harb. Perspect. Biol. 1, a003129 10.1101/cshperspect.a003129 20457567 PMC2882122

[2] Stepniak E, Radice GL, Vasioukhin V (2009) Adhesive and signaling functions of cadherins and catenins in vertebrate development. Cold Spring Harb. Perspect. Biol. 1, a002949 10.1101/cshperspect.a002949 20066120 PMC2773643

[3] Pokutta S, Weis WI (2007) Structure and mechanism of cadherins and catenins in cell-cell contacts. Annu. Rev. Cell Dev. Biol. 23, 237–261. 17539752 10.1146/annurev.cellbio.22.010305.104241

[4] Ohkubo T, Ozawa M (1999) p120 (ctn) binds to the membrane-proximal region of the E-cadherin cytoplasmic domain and is involved in modulation of adhesion activity. J. Biol. Chem. 274, 21409–21415. 10409703 10.1074/jbc.274.30.21409

[5] Thoreson MA, Anastasiadis PZ, Daniel JM, Ireton RC, Wheelock MJ, Johnson KR, et al (2000) Selective uncoupling of p120(ctn) from E-cadherin disrupts strong adhesion. J. Cell Biol. 148, 189–202. 10629228 10.1083/jcb.148.1.189 PMC2156209

[6] Ishiyama N, Lee SH, Liu S, Li GY, Smith MJ, Reichardt LF, et al (2010) Dynamic and static interactions between p120 catenin and E-cadherin regulate the stability of cell-cell adhesion. Cell. 141, 117–128. 10.1016/j.cell.2010.01.017 20371349

[7] Nanes BA, Chiasson-MacKenzie C, Lowery AM, Ishiyama N, Faundez V, Ikura M, et al (2012) p120-catenin binding masks an endocytic signal conserved in classical cadherins. J. Cell Biol. 199, 365–380. 10.1083/jcb.201205029 23071156 PMC3471230

[8] Davis MA, Ireton RC, Reynolds AB (2003) A core function for p120-catenin in cadherin turnover. J. Cell Biol. 163, 525–534. 14610055 10.1083/jcb.200307111 PMC2173649

[9] Xiao K, Allison DF, Buckley KM, Kottke MD, Vincent PA, Faundez V, et al (2003) Cellular levels of p120 catenin function as a set point for cadherin expression levels in microvascular endothelial cells. J. Cell Biol. 163, 535–545. 14610056 10.1083/jcb.200306001 PMC2173638

[10] Keirsebilck A, Bonné S, Staes K, van Hengel J, Nollet F, Reynolds A, et al (1998) Molecular cloning of the human p120 ctn catenin gene (CTNND1): expression of multiple alternatively spliced isoforms. Genomics. 50, 129–146. 9653641 10.1006/geno.1998.5325

[11] Reynolds AB, Daniel JM, Mo YY, Wu J, Zhang Z (1996) The novel catenin p120 cas binds classical cadherins and induces an unusual morphological phenotype in NIH3T3 fibroblasts. Exp. Cell Res. 225, 328–337. 8660921 10.1006/excr.1996.0183

[12] Fukumoto Y, Shintani Y, Reynolds AB, Johnson KR, Wheelock MJ (2008) The regulatory or phosphorylation domain of p120 catenin controls E-cadherin dynamics at the plasma membrane. Exp. Cell Res. 314, 52–67. 17719574 10.1016/j.yexcr.2007.07.024 PMC2211447

[13] Ozawa M, Kemler R (1990) Correct proteolytic cleavage is required for the cell adhesive function of uvomorulin. J. Cell Biol, 111, 1645–1650. 2211831 10.1083/jcb.111.4.1645 PMC2116240

[14] Ozawa M (2002) Lateral dimerization of the E-cadherin extracellular domain is necessary but not sufficient for adhesive activity. J. Biol. Chem. 277, 19600–19608. 11916976 10.1074/jbc.M202029200

[15] Koch AW, Farooq A, Shan W, Zeng L, Colman DR, Zhou MM (2004) Structure of the neural (N-) cadherin prodomain reveals a cadherin extracellular domain-like fold without adhesive characteristics. Structure. 2, 793–805. 10.1016/j.str.2004.02.034 15130472

[16] Posthaus H, Dubois CM, Laprise MH, Grondin F, Suter MM, Müller E (1998) Proprotein cleavage of E-cadherin by furin in baculovirus over-expression system: potential role of other convertases in mammalian cells. FEBS Lett. 438, 306–310. 9827567 10.1016/s0014-5793(98)01330-1

[17] Wahl JK, Kim YJ, Cullen JM, Johnson KR, Wheelock MJ (2003) N-cadherin-catenin complexes form prior to cleavage of the proregion and transport to the plasma membrane. J. Biol. Chem. 278, 17269–17276. 12604612 10.1074/jbc.M211452200

[18] Ozawa M, Kemler R (1992) Molecular organization of the uvomorulin-catenin complex. J. Cell Biol. 116, 989–996. 1734027 10.1083/jcb.116.4.989 PMC2289345

[19] Hinck L, Näthke IS, Papkoff J, Nelson WJ (1994) Dynamics of cadherin/catenin complex formation: novel protein interactions and pathways of complex assembly. J. Cell Biol. 125, 1327–1340. 8207061 10.1083/jcb.125.6.1327 PMC2290923

[20] Curtis MW, Johnson KR, Wheelock MJ (2008) E-cadherin/catenin complexes are formed cotranslationally in the endoplasmic reticulum/Golgi compartments. Cell Commun. Adhes. 15, 365–378. 10.1080/15419060802460748 18937087 PMC2742162

[21] Chen X, Kojima S, Borisy GG, Green KJ (2003) P120 catenin associates with kinesin and facilitates the transport of cadherin-catenin complexes to intercellular junctions. J. Cell Biol. 163, 547–557. 14610057 10.1083/jcb.200305137 PMC2173663

[22] Hernandez MV, Wehrendt DP, Arregui CO (2010) The Protein Tyrosine Phosphatase PTP1B Is Required for Efficient Delivery of N-Cadherin to the Cell Surface. Mol. Biol. Cell. 21, 1387–1397. 10.1091/mbc.E09-10-0880 20181825 PMC2854096

[23] Baki L, Marambaud P, Efthimiopoulos S, Georgakopoulos A, Wen P, Cui W, et al (2001) Presenilin-1 binds cytoplasmic epithelial cadherin, inhibits cadherin/p120 association, and regulates stability and function of the cadherin/catenin adhesion complex. PNAS. 98, 2381–2386. 11226248 10.1073/pnas.041603398 PMC30147

[24] Kizhatil K, Davis JQ, Davis L, Hoffman J, Hogan BL and Bennett V (2007) Ankyrin-G is a molecular partner of E-cadherin in epithelial cells and early embryos. J. Biol. Chem, 282, 26552–26561. 17620337 10.1074/jbc.M703158200

[25] Heisler FF, Lee HK, Gromova KV, Pechmann Y, Schurek B, Ruschkies L, et al (2014) GRIP1 interlinks N-cadherin and AMPA receptors at vesicles to promote combined cargo transport into dendrites. PNAS. 111, 5030–5035. 10.1073/pnas.1304301111 24639525 PMC3977296

[26] Zhao C, Slevin JT, Whiteheart SW (2007) Cellular functions of NSF: Not just SNAPs and SNAREs. FEBS lett. 581, 2140–2149. 17397838 10.1016/j.febslet.2007.03.032 PMC1948069

[27] Kojima S-I, Vignjevic D, Borisy GG (2004) Improved silencing vector co-expressing GFP and small hairpin RNA. Biotechniques 36, 74–79. 14740488 10.2144/04361ST02

[28] Sangwan V, Abella J, Lai A, Bertos N, Stuible M, Tremblay ML, et al (2011) Protein Tyrosine Phosphatase PTP1B Modulates Early Endosome Fusion and Trafficking of Met and EGF Receptor Tyrosine Kinases. J. Biol. Chem. 286, 45000–45013. 10.1074/jbc.M111.270934 22045810 PMC3247994

[29] Haj FG, Verveer PJ, Squire A, Neel BG, Bastiaens PI (2002) Imaging sites of receptor dephosphorylation by PTP1B on the surface of the endoplasmic reticulum. Science 295, 1708–1711. 11872838 10.1126/science.1067566

[30] Lázaro-Martínez JM, Rodríguez-Castellón E, Vega D, Monti GA, Chattah AK (2015) Solid-state Studies of the Crystalline/Amorphous Character in Linear Poly (ethylenimine hydrochloride) (PEI· HCl) Polymers and Their Copper Complexes. Macromolecules. 48, 1115–1125.

[31] Wu J, Mariner MA, Thoreson MA, Reynolds AB (1998) Production and characterization of monoclonal antibodies to the catenin p120^ctn^. Hybridoma 17, 175–183. 9627058 10.1089/hyb.1998.17.175

[32] Mo YY, Reynolds AB (1996) Identification of murine p120cas isoforms and heterogeneous expression of p120cas isoforms in human tumor cell lines.Cancer Research, 56, 2633–2640. 8653709

[33] Appenzeller-Herzog C, Hauri HP (2006) The ER-Golgi intermediate compartment (ERGIC): in search of its identity and function. J. Cell Sci. 119, 2173–2183. 16723730 10.1242/jcs.03019

[34] Ben-Tekaya H, Miura K, Pepperkok R, Hauri HP (2005) Live imaging of bidirectional traffic from the ERGIC. J. Cell Sci. 118, 357–367. 15632110 10.1242/jcs.01615

[35] Naydenov NG, Brown B, Harris G, Dohn MR, Morales VM, Baranwal S, et al (2012) A membrane fusion protein αSNAP is a novel regulator of epithelial apical junctions. PloS One. 7, e34320 10.1371/journal.pone.0034320 22485163 PMC3317505

[36] Zarelli VE, Ruete MC, Roggero CM, Mayorga LS, Tomes CN (2009) PTP1B dephosphorylates N-ethylmaleimide-sensitive factor and elicits SNARE complex disassembly during human sperm exocytosis. J. Biol. Chem. 284, 10491–10503. 10.1074/jbc.M807614200 19208619 PMC2667736

[37] Shi L, Zhang Q, Jiang X, Dai Y, Zhang CY, Zen K (2014) Sustained high protein-tyrosine phosphatase 1B activity in the sperm of obese males impairs the sperm acrosome reaction. J. Biol. Chem. 289, 8432–8441. 10.1074/jbc.M113.517466 24519936 PMC3961668

[38] Coppolino MG, Kong C, Mohtashami M, Schreibe AD, Brumell JH, Finlay BB, et al (2001) Requirement for N-Ethylmaleimide-sensitive Factor Activity at Different Stages of Bacterial Invasion and Phagocytosis. J. Biol. Chem. 276, 4772–4780. 11092884 10.1074/jbc.M007792200

[39] Dalal S, Rosser MFN, Cyr DM, Hanson PI (2004) Distinct Roles for the AAA ATPases NSF and p97 in the Secretory Pathway. Mol. Biol. Cell. 15, 637–648. 14617820 10.1091/mbc.E03-02-0097 PMC329284

[40] Anastasiadis PZ, Reynolds AB (2000) The p120 catenin family: complex roles in adhesion, signaling and cancer. J. Cell Sci. 113, 1319–1334. 10725216 10.1242/jcs.113.8.1319

[41] Ireton RC, Davis M, van Hengel J, Mariner DJ, Barnes K, Thoreson MA, et al (2002) A novel role for p120 catenin in E-cadherin function. J. Cell Biol. 159, 465–476. 12427869 10.1083/jcb.200205115 PMC2173073

[42] Giraudo CG, Rosales Fritz VM, Maccioni HJ (1999) GA2/GM2/GD2 synthase localizes to the trans-golgi network of CHO-K1 cells. Biochem. J. 342, 633–640. 10477274 PMC1220504

[43] Meng W, Takeichi M (2009) Adherens junction: molecular architecture and regulation. Cold Spring Harb. Perspect. Biol. 1, a002899 10.1101/cshperspect.a002899 20457565 PMC2882120

[44] Baum B, Georgiou M (2011) Dynamics of adherens junctions in epithelial establishment, maintenance, and remodeling. J. Cell Biol. 192, 907–917. 10.1083/jcb.201009141 21422226 PMC3063136

[45] Kowalczyk AP, Nanes BA (2012) Adherens Junction Turnover: Regulating Adhesion Through Cadherin Endocytosis, Degradation, and Recycling. (T. Harris, Ed.) Subcell. Biochem. 60, 197–222. 10.1007/978-94-007-4186-7_9 PMC4074012 22674073

[46] Shore EM, Nelson WJ (1991) Biosynthesis of the Cell Adhesion Molecule Uvomorulin (E-Cadherin) in Madin-Darby Canine Kidney Epithelial Cells. J. Biol. Chem. 266, 19672–19680. 1918074

[47] Chen YT, Stewart DB, Nelson WJ (1999) Coupling assembly of the E-cadherin/beta-catenin complex to efficient endoplasmic reticulum exit and basal-lateral membrane targeting of E-cadherin in polarized MDCK cells. J. Cell Biol. 144, 687–699. 10037790 10.1083/jcb.144.4.687 PMC2132940

[48] Kowalczyk AP, Reynolds AB (2004) Protecting your tail: regulation of cadherin degradation by p120-catenin. Curr. Opin. Cell Biol. 16, 522–527. 15363802 10.1016/j.ceb.2004.07.001

[49] Roczniak-Ferguson A, Reynolds AB (2003) Regulation of p120-catenin nucleocytoplasmic shuttling activity. J. Cell. Sci. 116, 4201–4212. 12953069 10.1242/jcs.00724

[50] Yanagisawa M, Kaverina IN, Wang A, Fujita Y, Reynolds AB, Anastasiadis PZ (2004) A novel interaction between kinesin and p120 modulates p120 localization and function. J. Biol. Chem. 279, 9512–9521. 14676216 10.1074/jbc.M310895200

[51] Teng J, Rai T, Tanaka Y, Takei Y, Nakata T, Hirasawa M, et al (2005) The KIF3 motor transports N-cadherin and organizes the developing neuroepithelium. Nat. Cell Biol. 7, 474–482. 15834408 10.1038/ncb1249

[52] Miyashita Y, Ozawa M (2007) A dileucine motif in its cytoplasmic domain directs β-catenin-uncoupled E-cadherin to the lysosome. J. Cell Sci. 120, 4395–4406. 18057030 10.1242/jcs.03489

[53] Nakamura T, Hayashi T, Mimori-Kiyosue Y, Sakaue F, Matsuura K, Iemura S, et al (2010) The PX-RICS-14-3-3zeta/theta complex couples N-cadherin-beta-catenin with dynein-dynactin to mediate its export from the endoplasmic reticulum. J. Biol. Chem. 285, 16145–16154. 10.1074/jbc.M109.081315 20308060 PMC2871483

[54] Troyanovsky RB, Klingelhöfer J, Troyanovsky SM (2011) α-Catenin contributes to the strength of E-cadherin–p120 interactions. Mol. Biol. Cell. 22, 4247–4255. 10.1091/mbc.E11-03-0250 21937720 PMC3216651

[55] Arregui CO, Pathre P, Lilien J, Balsamo J (2000) The nonreceptor tyrosine kinase fer mediates cross-talk between N-cadherin and beta1-integrins. J. Cell Biol. 149, 1263–1274. 10851023 10.1083/jcb.149.6.1263 PMC2175119

[56] Frangioni JV, Beahm PH, Shifrin V, Jost CA, Neel BG (1992) The nontransmembrane tyrosine phosphatase PTP-1B localizes to the endoplasmic reticulum via its 35 amino acid C-terminal sequence. Cell. 68, 545–560. 1739967 10.1016/0092-8674(92)90190-n

[57] Silverman JB, Restituito S, Lu W, Lee-Edwards L, Khatri L, Ziff EB (2007) Synaptic anchorage of AMPA receptors by cadherins through neural plakophilin-related arm protein AMPA receptor-binding protein complexes. J. Neurosci. 27, 8505–8516. 17687028 10.1523/JNEUROSCI.1395-07.2007 PMC6672939

[58] Naydenov NG, Harris G, Brown B, Schaefer KL, Das SK, Fisher PB, et al (2012) Loss of soluble N-ethylmaleimide-sensitive factor attachment protein α (αSNAP) induces epithelial cell apoptosis via down-regulation of Bcl-2 expression and disruption of the Golgi. J. Biol. Chem. 287, 5928–5941. 10.1074/jbc.M111.278358 22194596 PMC3285361

[59] Peter F, Wong SH, Subramaniam VN, Tang BL, Hong W (1998) Alpha-SNAP but not gamma-SNAP is required for ER-Golgi transport after vesicle budding and the Rab1-requiring step but before the EGTA-sensitive step. J. Cell Sci. 111, 2625–2633. 9701561 10.1242/jcs.111.17.2625

