# Supplementary material for: P120-Catenin Regulates Early Trafficking Stages of the N-Cadherin Precursor Complex
Source: PLoS One. 2016 Jun 2;11(6):e0156758. doi: 10.1371/journal.pone.0156758 (PMC4890775; doi:10.1371/journal.pone.0156758)

Figure A

HeLa cells

N-cadherin levels (endogenous vs exogenous)

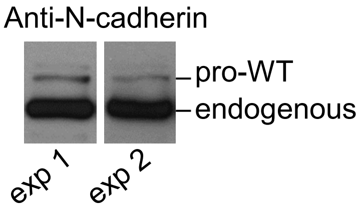

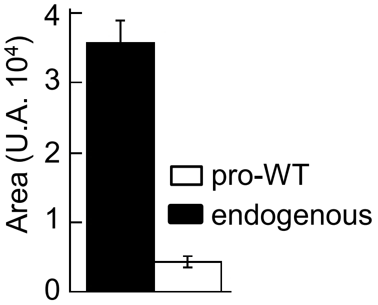

Figure B

HeLa cells

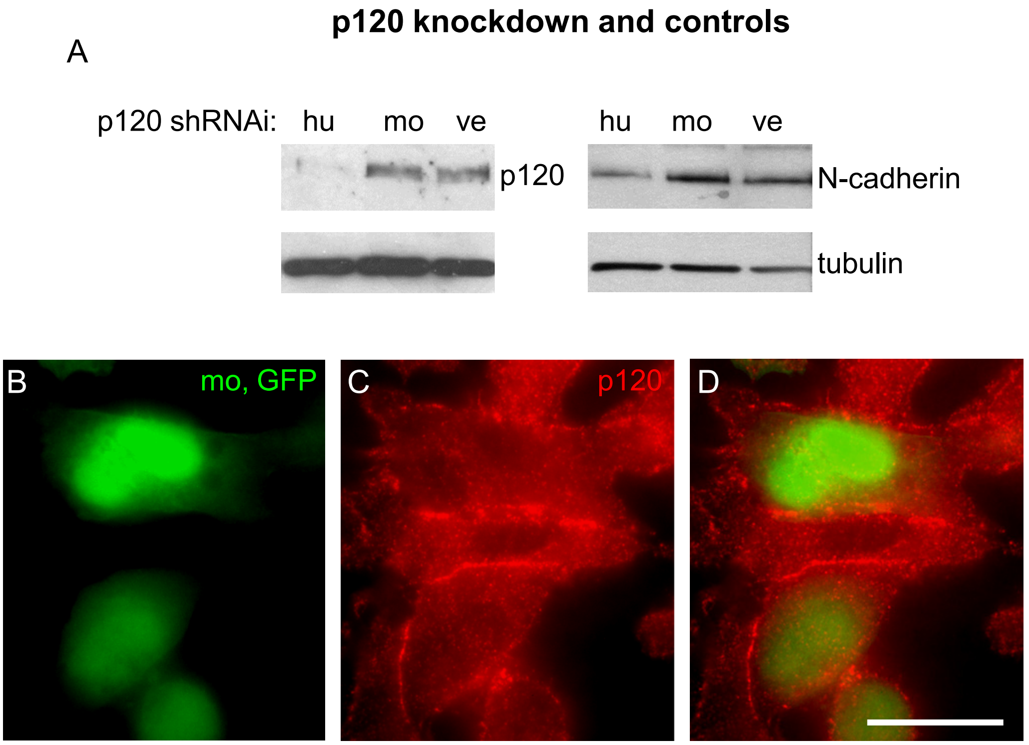

Supplement: S1 File — Figure A: Expression levels of pro-WT. The HA-N-cadherin-GFP (pro-WT) construct was transfected in HeLa cells and the expression levels were analyzed by Western blotting with anti-N-cadherin, to allow for the direct comparison with endogenous N-cadherin levels. Due to the GFP fusion, pro-WT migrates slower than endogenous N-cadherin. Under our detection conditions essentially all ectopic and endogenous N-cadherin correspond to mature, processed proteins. Representative blots of two independent experiments are shown at the left. Quantification of the bands is shown in the graph. Bars represent means ± S.E.M. from five experiments. Figure B: Effect of p120 shRNAi targeting. (A) Western blotting analysis of HeLa cells transfected with empty pG-Shin2 vector (ve), the vector encoding the human p120 targeting sequence (hu), or the equivalent mouse p120 targeting sequence (mo). Blots were probed with anti-p120 (clone 6H11), anti-N-cadherin, and anti-tubulin. Only the human p120 targeting sequence showed a marked effect on p120 expression. A representative blot of 3 independent experiments is shown. (B-D) HeLa cells expressing the control, mouse p120 targeting sequence. Immunofluorescence with anti-p120 reveals similar p120 levels (red signal) in transfected (GFP positive cells) and non transfected cells. Scale bar in (D), 35 μm. (PDF) [file pone.0156758.s001.pdf]
